# Prevalence and antibiogram of *Staphylococcus aureus* isolates in bovine raw milk from small-scale farmers in Magu district, Mwanza, Tanzania

**DOI:** 10.1186/s12917-025-05184-y

**Published:** 2026-01-06

**Authors:** Doris R. Ngassa, Alice S. Lakati, Mariam M. Mirambo

**Affiliations:** 1 Department of Community Health, Amref International University, P.O. Box 27691-00506, Nairobi, Kenya; 2 Department of Microbiology and Immunology, Catholic University of Health and Allied Sciences, P.O. Box 1464, Mwanza, Tanzania

**Keywords:** AMR, Bovine raw milk, MDR, MRSA, *Staphylococcus aureus*

## Abstract

**Background:**

*Staphylococcus aureus*, a common cause of foodborne illnesses, may be transmitted from cows to humans through contaminated raw milk. Limited data exist on livestock-associated *Staphylococcus aureus* (LA-*S. aureus*) infections in Tanzania’s bovine supply chain. This study assessed the prevalence and antibiogram of *Staphylococcus aureus* in cows’ raw milk, from small-scale farmers in Magu district, Mwanza, Tanzania.

**Methods:**

A cross-sectional study was conducted between April 2023 and June 2024. A total of 410 non-repetitive raw milk samples were collected from cows of small-scale farmers. Milk samples were processed to isolate *Staphylococcus aureus* as per laboratory standard procedures. Drug susceptibility was established with the Kirby-Bauer disk diffusion method following the Clinical and Laboratory Standards Institute (CLSI) guidelines, 2022. R software was used to analyze percentages and proportions of the data for all variables.

**Results:**

The prevalence of *Staphylococcus aureus* in raw milk from small-scale farmers was 23.9% (98/410), with 16.3% (16/98) being methicillin-resistant *Staphylococcus aureus* (MRSA). The resistance proportions were penicillin 45.9% (45/98), tetracycline 33.7% (33/98), erythromycin 21.4% (21/98), cefoxitin 16.3% (16/98), clindamycin 6.1% (6/98), trimethoprim-sulfamethoxazole 6.1% (6/98), gentamicin 3.1% (3/98), and ciprofloxacin 1.0% (1/98). Of the 98 *Staphylococcus aureus* isolates, 20 (20.4%) were multidrug resistant (MDR) defined as resistance to three or more antibiotics.

**Conclusion:**

The prevalence of *Staphylococcus aureus* was high with a significant proportion of isolates being MRSA, in raw milk from small-scale farmers. High proportions of antibiotic resistance and MDR patterns underscore the urgent need for improved antimicrobial stewardship and the promotion of responsible antibiotic use in dairy farming.

**Supplementary Information:**

The online version contains supplementary material available at 10.1186/s12917-025-05184-y.

## Background

Over the past five decades, *Staphylococcus aureus* has gained attention due to its ability to adapt to antibiotic pressure, leading to a high burden of antibiotic resistance [1]. The current statistics indicate a global linkage of 4.95 million deaths to antimicrobial resistance (AMR), with 1.27 million solely from AMR and 366,000 specifically from Eastern Sub-Saharan African countries [2]. By 2050, it is projected that AMR could lead to 10 million deaths annually if effective countermeasures are not implemented [3, 4]. Failure to address this issue with urgency could lead to a major global public health challenge [4].

*Staphylococcus aureus* is a widely prevalent Gram-positive bacterium, that is implicated in various infections across human and animal populations, ranging from simple abscesses to severe necrotizing pneumonia and cow mastitis which is a significant issue in dairy farming worldwide [5–7]. The emergence of methicillin-resistant *Staphylococcus aureus* (MRSA), resistant to β-lactam antibiotics, poses a significant challenge in both clinical and veterinary settings due to limited treatment options [1, 8]. These strains not only jeopardize animal welfare and dairy production economies but also pose serious public health risks, necessitating intensified efforts to combat their spread and impact [9–11].

The rapid development of AMR stems from various factors such as improper antibiotic use driven by diagnostic challenges, patient demand, and financial incentives [12]. Treating MRSA infections in both humans and animals remains challenging due to limited treatment options, hence emphasizing milking hygiene, boiling, and pasteurization to reduce transmission risks [13, 14]. AMR is exacerbated by the non-therapeutic use of antibiotics such as for growth promotion or prophylaxis rather than for treating infections among farmers due to chaotic animal health services, coupled with the exposure of livestock workers to MRSA-carrying animals like pigs and cattle, alongside global mobility, which facilitates the spread of antibiotic-resistant pathogens [15, 16].

Research across various countries, including Turkey [16], Tanzania [17] and Indonesia [18], has highlighted *Staphylococcus aureus* prevalence in raw milk samples, with rates ranging from 15% to 72% and MRSA prevalence of 2.9% to 20%. Antibiotic resistance patterns among *Staphylococcus aureus* isolates have shown diverse profiles, with a notable presence of multidrug resistance (MDR) strains [17]. The efforts to improve milk quality and enhance surveillance, especially in regions like Africa where data on LA-*S. aureus* prevalence and AMR are limited, are crucial to addressing the challenges related to the spread of resistant strains, suboptimal milk quality, and the lack of comprehensive surveillance systems [17–21].

Addressing the existing knowledge gaps is essential, particularly regarding the transmission dynamics of LA*-S. aureus* between humans and animals and its implications for public health. This study aimed at assessing the prevalence and antibiogram of *Staphylococcus aureus* in cows’ raw milk, from small-scale farmers in Magu district, Mwanza, Tanzania, and inform on targeted interventions that can effectively mitigate its spread.

## Methods

### Study location and design

**Fig. 1:**
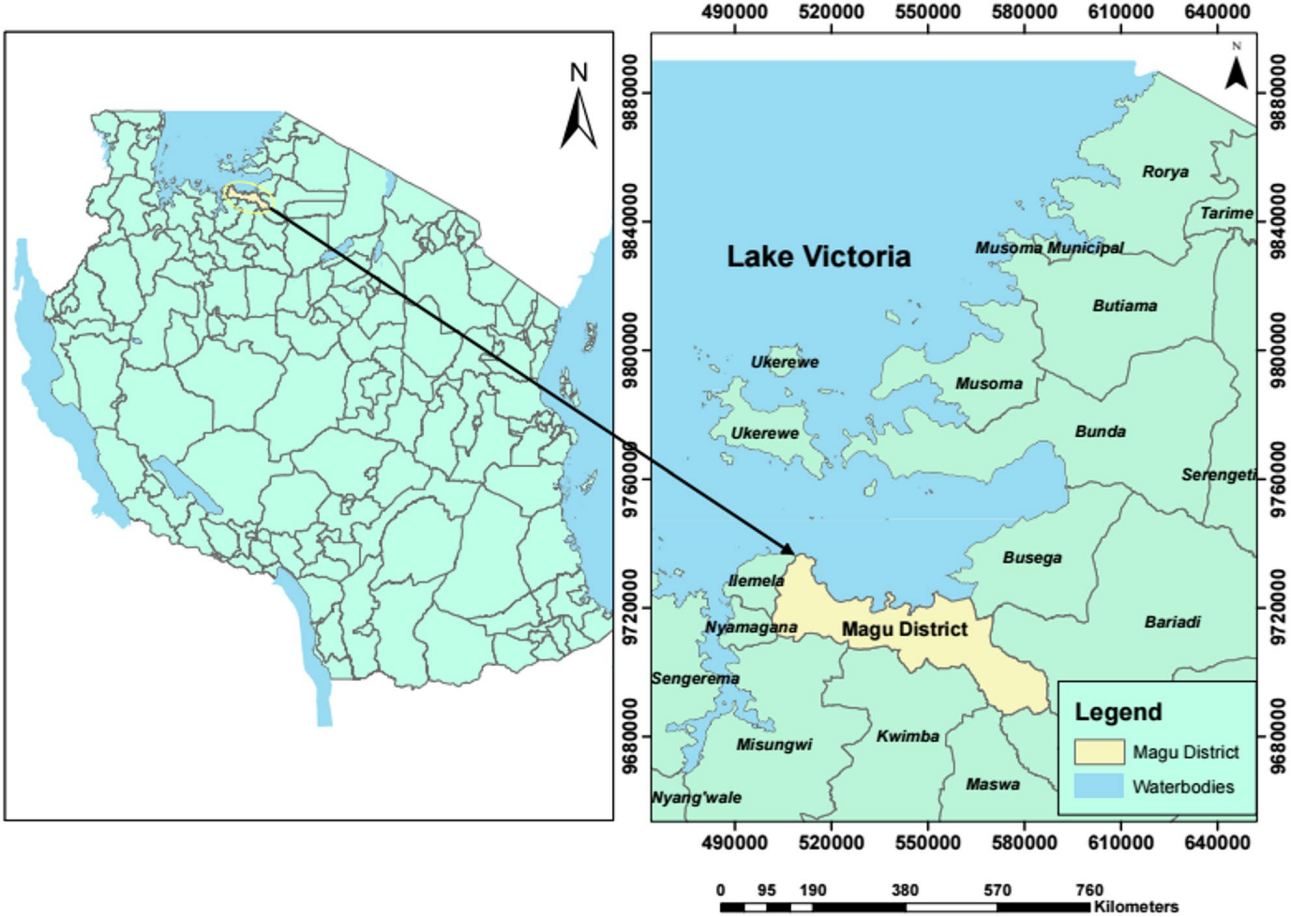
A map showing the study area, Magu district, Mwanza, Tanzania

A cross-sectional study was conducted in the Magu district, Mwanza, Tanzania, between April, 2023 and June 2024. The city of Mwanza is located on the southern shores of Lake Victoria between 1200 and 1400 meters above sea level. Magu district is 63.6 kilometers from the city of Mwanza and borders Ukerewe district to the north, Kwimba and Misungwi districts to the south, Ilemela district to the west, and Busega district to the east. Magu district lies between longitudes 33° and 30° east of Greenwich, latitudes 20°10’ and 2°50’ south of the Equator, Fig. 1. Magu district has an average temperature of 29 °C and an estimated population of 421,119 residents [22].

The targeted population was small-scale farmers, identified as pastoralists or agro-pastoralists with indigenous or crossbred lactating cows. These farmers operated on small landholdings, managing cattle in either intensive, or extensive systems. Milk was produced for household use or sold in local markets. In Magu, as in other parts of Tanzania, much of the raw milk is consumed without pasteurization or boiling, with up to 65% consumed raw in Arusha, which increases the risk of *Staphylococcus aureus* and MRSA contamination [20, 23]. Further research in Magu District is needed to assess the prevalence of these pathogens and the associated public health risks.

### Sampling and sample collection

The sample size was determined using the formula by Thrusfield, 2005 [24], (n = Zα² * p(1 − p)/e²), aiming for a 95% confidence level with a 5% margin of error and assuming a 50% predicted prevalence of *Staphylococcus aureus*. As a result, a total of 410 non-repetitive raw milk samples were collected from 48 herds owned by small-scale farmers. These farmers typically managed herds ranging from 4 to 17 cattle, relying on family labor and traditional practices. Wards, defined as local administrative subdivisions of districts in Tanzania, and households were conveniently selected based on the availability of farmers and their willingness to participate in the study. All lactating cows on the sampling day were included. The selected farms reflect small-scale operations in Magu district and similar regions in Tanzania, where hand-milking is a common practice.

The milk samples collected were primarily consumed by the household or sold in local markets. Aseptic techniques were employed during milk collection using disinfected gloved hands. Prior to collection, each teat was carefully washed with warm water, dried, and then disinfected with 70% alcohol, focusing especially on the teat ends. To reduce contaminants, the first few streams of milk were discarded. Approximately 12 mL of milk was pooled from all four teats, with equal volumes collected from each quarter, into sterile falcon tubes (CITOTEST^®^ Ltd., Halmen City, Hants, China) for analysis. The milk samples were then transported in a cool box and stored at a temperature of 4 °C. Processing of the samples for bacteriological examination took place the following day at the Tanzania Veterinary Laboratory Agency (TVLA), Mwanza, Tanzania.

### Bacteria isolation and identification

Bovine raw milk samples were processed for bacterial isolation and identification using conventional methods as described previously [25]. Initially, 410 raw milk samples were inoculated onto blood agar (OXOID^®^ Ltd., Basingstoke, Hants, United Kingdom) plates supplemented with 5% sheep blood and incubated overnight at 37 °C to isolate *Staphylococcus aureus* colonies based on the gram stain, colony morphology, and hemolytic patterns. Subsequently, identified colonies were sub-cultured on Mannitol salt agar (LIOFILCHEM^®^, Roseto degli Abruzzi, Italy) to ensure purity and then subjected to catalase, slide, and tube coagulase test to confirm *Staphylococcus aureus* [25].

### Antimicrobial susceptibility test

*Staphylococcus aureus* isolates were tested for susceptibility using the Kirby-Bauer disk diffusion method, as outlined by the Clinical and Laboratory Standards Institute (CLSI) guidelines, 2022 [26], for the selected antibiotics used in Tanzania, both by humans and by animals as previously reported [27]. Antibiotic disks (OXOID^®^ Ltd., Basingstoke, Hants, United Kingdom) used were cefoxitin (30 µg), ciprofloxacin (5 µg), clindamycin (2 µg), erythromycin (15 µg), gentamicin (10 µg), penicillin (10 µg), tetracycline (30 µg), and trimethoprim/sulfamethoxazole (1.25/23.75 µg). A sterile wire loop was employed to pick three pure *Staphylococcus aureus* colonies, which were suspended in normal saline to produce a turbid bacterial suspension equivalent to 0.5 of the McFarland standard.

The bacterial suspension was evenly spread onto Muller Hinton Agar (OXOID^®^ Ltd., Basingstoke, Hants, United Kingdom) using the lawn method, which involved swabbing the entire surface of the agar to ensure uniform bacterial distribution. The plate was allowed to air dry before antibiotic disks were placed on the surface. Antibiotic disks were then added, and the plate was incubated for 16 to 18 h at 37 °C [28]. A metric ruler in millimeters (mm) was used to measure disc inhibitory zone diameter and interpreted as per CLSI, 2022 [26],. MRSA isolates were defined based on resistance to a 30 µg cefoxitin disk. MDR *Staphylococcus aureus* was defined as isolates acquired non-susceptibility to at least one agent in three or more antimicrobial categories [29].

### Data analysis

Data were entered into and stored in a Microsoft Excel spreadsheet, then reviewed for missing values, duplicates, and inconsistencies by cross-referencing original records. Descriptive analysis was performed using R software version 4.3.1. The prevalence of *Staphylococcus aureus* and MRSA was determined by calculating the proportion of positive isolates relative to the total number of samples examined. Antibiogram of *Staphylococcus aureus* were assessed by evaluating the proportion of *Staphylococcus aureus* isolates resistant to tested antibiotics and multiple antibiotics for MDR.

## Results

### Prevalence of *Staphylococcus aureus*

Ninety-eight out of four hundred and ten milk samples (98/410) were *Staphylococcus aureus* positive, constituting a prevalence of 23.9% (95% CI, 19.8–28.0). Among these, 16 *Staphylococcus aureus* isolates were identified as MRSA, representing an MRSA proportion of 16.3% (95% CI: 9.9–22.7) and an overall prevalence of 3.9% (95% CI: 2.0–5.8) among the total milk samples.

### Antibiogram of *Staphylococcus aureus*

The antimicrobial profile of *Staphylococcus aureus* showed notable resistance rates of the isolates to penicillin 45.9% (45/98), tetracycline 33.7% (33/98), erythromycin 21.4% (21/98), cefoxitin 16.3% (16/98) and clindamycin 6.1% (6/98). Intermediate susceptibility was predominant for clindamycin 14.3% (14/98), erythromycin 11.2% (11/98) and ciprofloxacin 7.1% (7/98). The most sensitive antibiotics were gentamicin 94.9% (93/98), ciprofloxacin 91.8% (90/98), and trimethoprim-sulfamethoxazole 90.8% (89/98), indicating their efficacy against the studied isolates, Table 1.

**Table 1:** Antibiogram of *Staphylococcus aureus*

Antimicrobial	Antimicrobial Susceptibility Test (AST) Results		
	%Susceptible (*n* = 98)	%Intermediate (*n* = 98)	%Resistant (*n* = 98)
Cefoxitin (30 µg)	82(83.7)	0(0)	16(16.3)
Ciprofloxacin (5 µg)	90(91.8)	7(7.1)	1(1.1)
Clindamycin (2 µg)	78(79.6)	14(14.3)	6(6.1)
Erythromycin (15 µg)	66(67.3)	11(11.2)	21(21.4)
Gentamycin (10 µg)	93(94.9)	2(2.0)	3(3.1)
Penicillin (10 µg)	53(54.1)	0(0)	45(45.9)
Tetracycline (30 µg)	59(60.2)	6(6.1)	33(33.7)
Tri-Sulfa (1.25/23.75 µg)	89(90.8)	3(3.1)	6(6.1)

*n* Number of Isolates, % Percentage, *µg* Microgram

### MDR patterns of *Staphylococcus aureus* and MRSA

Of the 98 *Staphylococcus aureus* isolates, 20 (20.4%) displayed MDR, defined as resistance to three or more antibiotics representing an overall MDR prevalence of 4.9% (95% CI: 2.9%–7.5%) of the total milk samples. Among the *Staphylococcus aureus* isolates, 11.2% (11/98) were identified as multidrug-resistant MRSA, Table 2.

**Table 2:** MDR patterns of *Staphylococcus aureus* and MRSA

Antibiotic pattern	Number of Staphylococcus aureus	Number of MRSA	Number of antibiotic classes
Clin-Ery-Pen	1	-	3
Ery-Gen-Pen	2	-	3
Ery-Pen-Tet	4	-	3
Fox-Clin-Pen	1	1	3
Fox-Ery-Pen	1	1	3
Fox-Gen-Pen	1	1	3
Fox-Pen-Tet	1	1	3
Fox-Pen-SXT	1	1	3
Ery-Pen-Tet-SXT	2	-	4
Fox-Clin-Ery-Pen	1	1	4
Fox-Ery-Pen-Tet	2	2	4
Fox-Cip-Ery-Pen-SXT	1	1	5
Fox-Clin-Ery-Pen-SXT	1	1	5
Fox-Ery-Pen-Tet-SXT	1	1	5
Total	**20**	**11**	**-**

*Fox* Cefoxitin, *Cip* Ciprofloxacin, *Clin* Clindamycin, *Ery* Erythromycin, *Gen* Gentamycin, *Pen* Penicillin, *Tet* Tetracycline, *SXT* Trimethoprim-Sulfamethoxazole

There were no MRSA isolates with MDR of the following combinations: Clin-Ery-Pen, Ery-Gen-Pen, Ery-Pen-Tet, Ery-Pen-Tet-SXT.

## Discussion

The availability of microbiological data is crucial for understanding the prevalence and antibiotic resistance patterns of pathogens, which is essential for developing control measures. In Tanzania, many people rely on readily available raw milk supplied by local small-scale farmers [20]. The widespread consumption of raw milk raises concerns about contamination risks, particularly from *Staphylococcus aureus*, a pathogen linked to foodborne illnesses [30]. However, there is limited data on the microbiological quality of milk produced by small-scale farmers in the region. This study documents a high prevalence of *Staphylococcus aureus* and MRSA in bovine raw milk from small-scale farmers in the Magu district, Mwanza, Tanzania, and provides an antibiogram to inform on antibiotic use.

In this study, nearly a quarter (23.9%; 95% CI: 19.8–28.0) of bovine raw milk samples were culture-positive for *Staphylococcus aureus*, indicating poor quality of the raw milk produced which can be harmful to the health of consumers. The milk samples were collected from small-scale farmers in the same area, suggesting that contamination could be related to similar milk production systems in the locality [31]. The prevalence of *Staphylococcus aureus* in this study is comparable to previous studies in Tanzania [17] and Ethiopia [30, 32, 33], which reported prevalence rates ranging from 21.5% to 24.9%. However, the current prevalence of *Staphylococcus aureus* is higher than 15% previous reported in Mbeya, Tanzania [34], but lower than 55% to 72.5% previous reported in Indonesia [18], Mozambique [35], and China [36]. These variations may be attributed to several factors, including differences in sample selection methods, study design, laboratory identification methods, and sample handling procedures, which commonly influence bacterial detection rates in milk studies. The presence of subclinically infected cows and regional differences in farm management and hygiene practices may also contribute to the observed variation.

Meanwhile, antibiotic resistance in bacteria is a natural survival mechanism, but it can have harmful consequences for society [37]. The resistant MRSA strains can enter the food chain and contribute to the spread of antibiotic resistance strains in humans, which is a global concern. In this study, 3.9% (95% CI: 2.0–5.8) of bovine raw milk samples were identified as MRSA, indicating presence of MRSA in the bovine milk supply chain. The prevalence of MRSA in this study is comparable to previous study in Ethiopia [32] which reported prevalence rate of 4%. However, the current prevalence of MRSA is higher than previous reported in Morogoro, Tanzania [19] but lower than 35.7% previous reported in Egypt [6]. These variations may be attributed to factors like antibiotic misuse, which drives the emergence and persistence of MRSA strains.

*Staphylococcus aureus* is known for its resistance to the penicillin group of antibiotics, primarily due to beta-lactamase production and the widespread use of antibiotics in the region [17]. Penicillin displayed the highest resistance rate of all antibiotics tested in this study, with a 45.9% resistance rate, which is comparable to 49.5% resistance rate previous reported in Algeria [38]. This similarity might be attributed to previous exposure with *Staphylococcus aureus* and the use of similar antimicrobial susceptibility testing methods, which enhance comparability. However, the resistance rate observed in this study is higher than the 23.8% previous reported in Mbeya, Tanzania [34], but lower than the 92% to 95% resistance rates previous reported in Tanzania [19] and Ethiopia [30, 32, 39]. These variations may be attributed to the presence of inherent resistance properties of the *Staphylococcus aureus* strains prevalent in the localities.

In addition to penicillin, the antibiogram of *Staphylococcus aureus* isolates for other tested antibiotics were: 33.7%, 21.4%, 16.3%, 6.1%, 6.1%, 3.1%, and 1.1% for tetracycline, erythromycin, cefoxitin, clindamycin, trimethoprim-sulfamethoxazole, gentamicin and ciprofloxacin, indicating a diverse range of resistance among the tested isolates. The current study’s findings on sensitivity to ciprofloxacin, gentamicin, and trimethoprim-sulfamethoxazole are comparable to previous reports in Turkey [16], Tanzania [19], and Ethiopia [40]. This similarity may be attributed to the broad-spectrum nature of these antibiotics. However, resistance of *Staphylococcus aureus* to other antibiotics, such as clindamycin, erythromycin, and tetracycline, was higher in previous studies in Turkey [16], China [36], and Ethiopia [40]. These variations could be explained by differences in the frequency of antibiotic use and regional antibiotic policies, such as the high use of tetracycline in veterinary medicine as previously reported in Indonesia [18] and Kenya [41]. The variability in AMR patterns of *Staphylococcus aureus* isolates across different geographical locations reflects the importance of regional context in understanding AMR trends.

Furthermore, the emergence of MDR *Staphylococcus aureus* poses a serious threat to effective treatment options, making infections harder to treat, prolonging illness, and increasing the risk of complications. In this study, 20.4% (20/98) of *Staphylococcus aureus* isolates exhibited MDR patterns, indicating resistance to multiple antibiotic classes. The MDR proportion observed in the current study is higher compared to previous reported in Tanzania [17]. Although both studies used a similar MDR categorization method, the differences in findings may be attributed to variations in antibiotic exposure patterns. However, only 11 out of 16 MRSA isolates were classified as MDR, demonstrating that MRSA status does not necessarily imply resistance to other antibiotic classes beyond β-lactams which is comparable to previous study in Ethiopia [42]. MRSA strains are primarily resistant to β-lactams, but may still exhibit susceptibility to other classes of antibiotics, depending on antibiotic used.

Given the absence of a well-structured distribution system for antimicrobial drugs marked by insufficient veterinary involvement and unregulated over the counter sales, farmers often obtain and administer antibiotics without proper prescription or guidance as previously reported [43]. Consequently, the emergence of more drug-resistant strains of *Staphylococcus aureus* may result from drug misuse within the livestock production chain. Further studies on the epidemiology of *Staphylococcus aureus* and the occurrence of AMR and MDR in Tanzania are strongly encouraged. Moreover, this study confirms the presence of *Staphylococcus aureus* and MRSA in the bovine milk supply chain in Tanzania, highlighting a potential risk to both livestock production and public health. These findings provide valuable evidence for policymakers and regulatory bodies, such as the Tanzania Dairy Board (TDB), to strengthen milk value chain monitoring and antimicrobial stewardship. As previously reported in Tanzania, only 2% of vendors in held a TDB license [44]. Establishing well-regulated milk collection and distribution systems, alongside stringent control of antibiotic sales and usage, is essential to ensure that milk produced meets safety and quality standards while minimizing the spread of antibiotic-resistant bacteria. Additionally, promoting hygienic milking practices and responsible antibiotic use at the farm level is critical to achieving these goals.

### Limitations

This study was limited by its geographic scope, sample size, and time frame. Expanding future investigations to multiple districts and conducting sampling across different seasons would provide more representative data. Additionally, this study did not assess farm management, milking hygiene, or mastitis prevention practices, which are key factors influencing the spread and persistence of *Staphylococcus aureus*, a zoonotic pathogen. Including such variables, together with molecular characterization of isolates, would enhance understanding of transmission dynamics and resistance mechanisms of *Staphylococcus aureus* and MRSA.

## Conclusion

Nearly a quarter of bovine raw milk samples from the Magu district have been found to contain *Staphylococcus aureus* bacteria, with MRSA accounting for a considerable proportion. This high prevalence underscores the need for further research on the occurrence, AMR, and MDR patterns of *Staphylococcus aureus* in Tanzania. Such studies are important for demonstrating the need to improve quality raw milk production and antibiotic stewardship. As evidenced by a previous study in Tabriz, Iran, MRSA contamination can still occur post-pasteurization and reach consumers, necessitating strict control measures across the milk supply chain [45]. The country through the dairy board, veterinary, and public health departments should take into consideration (i) strengthening the milk supply chain to reduce milk contamination and associated health risks from *LA-S. aureus* (ii) conducting and evaluating awareness campaigns on AMR in dairy-producing communities, and (iii) promoting responsible antibiotic use in dairy farming by implementing regulations that restrict antibiotic use to therapeutics. Furthermore, researches on the molecular characterization of *Staphylococcus aureus* and MRSA are recommended [17]. This information is important in understanding the specific *Staphylococcus aureus* strains and resistance mechanisms for monitoring evolving and persistent strains.

## Supplementary Information

Supplementary Material 1.

## Data Availability

The data sets used and/or analyzed are available from the corresponding authors on a reasonable request.

## Abbreviations

AMR: Antimicrobial resistance
CLSI: Clinical and Laboratory Standards Institute
LA-*S. aureus*: Livestock Associated *Staphylococcus aureus*
MDR: Multidrug Resistance
MRSA: Methicillin-Resistant *Staphylococcus aureus*
